# Women's Satisfaction with Abortion Care and Associated Factors in Public Health Facilities of Mojo Town, East Ethiopia

**DOI:** 10.1155/2023/4726878

**Published:** 2023-03-27

**Authors:** Tola Oda, Midekso Sento, Atoma Negera

**Affiliations:** ^1^Clinician at Meki Hospital, Oromia Regional State, Eastern Ethiopia, Ethiopia; ^2^Anatomy Course Unit, Biomedical Department, Adama Hospital Medical College, Eastern Ethiopia, Ethiopia; ^3^Nursing Department, Health Science College, Mattu University, Southwest Ethiopia, Ethiopia

## Abstract

**Background:**

Client satisfaction is an important and commonly used indicator for measuring the quality of health care as it affects clinical outcomes, patient retention, and medical malpractice claims. To limit unintended pregnancies and avoid repeated abortions promoting abortion care services is crucial. In Ethiopia, problems related to abortion were neglected, and access to quality abortion care was very limited. Similarly, information related to abortion care service, particularly clients' satisfaction, and associated factors are limited in the study area that the study will be going to fill.

**Methods:**

A facility-based cross-sectional study design was employed on 255 women who came for abortion service in public health facilities of Mojo town and were included consecutively. The data was coded and entered into Epi info version 7 software and exported to SPSS version 20 for analysis. Bivariable and multivariable logistic regression models were applied to identify the associated factors. Model fitness and multicollinearity were checked by using the Hosmer-Lemeshow goodness of fit test and the variance inflation factor (VIF). Adjusted odds ratios and their 95% confidence were reported.

**Results:**

A total of 255 study subjects were included in this study with a 100% response rate. The study depicted that 56.5% (95% CI: 51.3, 61.7 of the clients were satisfied with abortion care service. Having college and above educational level (AOR: 0.27; 95% CI: (0.14, 0.95), employee occupation (AOR: 1.86; 95% CI: (1.41, 2.93), medical abortion as a type of uterine evacuation (AOR: 3.93; 95% CI: (1.75, 8.83), and natural method of family planning users (AOR: 0.36; 95% CI: (0.08, 0.60) were factors associated with women's satisfaction.

**Conclusion:**

The overall satisfaction towards abortion care was considerably lower. Waiting time, cleanness of rooms, lack of laboratory service, and availability of service providers are mentioned factors for client dissatisfaction.

## 1. Introduction

Client satisfaction is an important and commonly used indicator for measuring the quality of health care as it affects clinical outcomes, patient retention, and medical malpractice claims. Evaluating the clients' satisfaction with health services is clinically relevant, as satisfied patients are more likely to comply with treatment, take an active role in their care, continue using medical care services, and recommend centers' services to others. A satisfied patient will recommend centers' services expressing their satisfaction to four or five people, while a dissatisfied patient will complain to twenty or more [1–3].

The quality of reproductive health care is critical in determining whether the service meets clients' expectations or not: for instance, they have choices of services, get accurate and complete information, technically competent care, good interaction with providers, continuity of care, and the constellation of related services [4]. Furthermore, healthcare providers' respectful treatment, patience, concern, and attentiveness have a crucial impact on patient satisfaction [5]. On the other hand, dissatisfied clients with medical services do not adhere to the advice of providers; they may not return for follow-up visits and ultimately compromise their health which may play a significant role in increasing maternal mortality and morbidity, as well as increases unsafe abortions [6, 7].

The World Health Organization estimates that, worldwide, almost 20 million unsafe abortions take place each year, and nearly 5.5 million African women have unsafe abortions. In Eastern Africa, it is estimated that 18% of all maternal deaths are the result of complications of poorly performed abortions. 80,000 maternal deaths per year are thought to be due to abortion complications, accounting for about 13% of all maternal deaths in the world [8, 9]. Each year, approximately 500,000 maternal deaths had occurred worldwide. Among 99% of these deaths occur in developing countries from which sub-Saharan Africa accounts for 50% of the maternal death burden [10]. Ethiopia is one of the countries with the highest maternal mortality ratio which is estimated at 412/100,000 live births [11]. The main contributing factors to this high death include unsafe abortion, among others [12]. Several studies in Ethiopia indicated that unsafe abortion may account for up to 25-35% of maternal deaths [13–15].

Comprehensive postabortion care is identified as an important intervention to treat complications resulting from miscarriage and unsafe abortion, reduce the incidence of repeat unplanned pregnancy, and decrease the incidence of repeat abortion [16]. According to WHO report, approximately,qualitymaternal death worldwide are abortion related and 5 million women aresuffered to complication of unsafe abortion and 99% of mortality and morbidity occurring developing countries [17]. However, access to quality, legalized, safe abortion services, quality family planning services, sexual education, and treatment of abortion complications can prevent maternal mortality and morbidity [8]. In sub-Saharan Africa, especially in Ethiopia, problems related to abortion were neglected, and access to quality abortion care was very limited [18–20]. Moreover, very little was known about the women's satisfaction with the abortion service provided. Therefore, this study is aimed at assessing client satisfaction with abortion services and identifying associated factors in Mojo town, Ethiopia.

## 2. Methods

### 2.1. Study Area

The study was conducted in Mojo town, which is located about 68 km east of Addis Ababa, (the capital city of Ethiopia). Mojo town's health institutions serve a large size of the population from the Oromia region and some parts of the Amhara region. In the town, there are three governmental health institutions (two health centers and one hospital). According to the town health bureau, all health facilities provide abortion care services.

### 2.2. Study Design and Period

The facility-based cross-sectional study design was conducted from September 1 to December 30, 2020.

### 2.3. Populations

#### 2.3.1. Source Population

All women who had/have abortion service in public health facility of Mojo town.

#### 2.3.2. Study Population

All women who received abortion services in the selected public health facility of Mojo town.

#### 2.3.3. Exclusion Criteria

Eligible women who were ill and unable to respond at the time of the study were excluded. The status of the above conditions was determined based on self-reports and observation.

### 2.4. Sample Size and Sampling Procedures

#### 2.4.1. Sample Size Determination

The sample size of the study participants for the first objective is based on a single population proportion, considering the following assumptions: 76.3% the proportion of women satisfied with abortion service [21], 95% confidence level (*Z*_*α*/2_ = 1.96), and margin of error calculated from a previous study, (*d* = 0.05), and with the addition of possible 10% nonresponse, the final sample size was 232 + 23 = 255.

The sample size of the study participants for the second objective (factors) was determined by using the Epi-info version7 (StatCalc) with the following assumptions; 95% confidence interval, % of nonexposed (*p*1) = proportion of unsatisfied clients with the educational status of college and above which was 23.7% [22], % of exposed (*p*2) = proportion of satisfied client with the educational status of college and above which was 44.4% [13]; level of required study power was 80%, ratio 1 : 1.

Using EPI-Info version 7, the total sample size was calculated as 182. The largest sample size of 255 was taken to fulfill all specific objectives.

#### 2.4.2. Sampling Procedure

**Figure 1:**
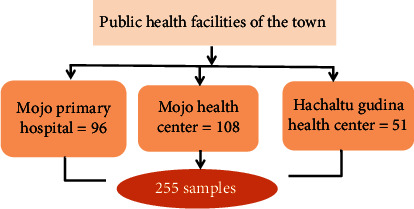
Schematic presentation of sampling procedures for women's satisfaction on abortion care and associated factors in public health facilities of Mojo town.

There are one public hospital and two health centers. All these health institutions provide abortion services and were included in the study. All clients who visited each health facility within the study period were included consecutively till the sample size fills (Figure 1).

### 2.5. Data Collection Tool and Procedure

#### 2.5.1. Data Collection Tool

Semistructured questionnaires were adapted from similar studies conducted in Ethiopia [16, 18, 21–23] and checked for internal consistency by Cronbach's Alpha. The questionnaire measures sociodemographic characteristics, reproductive health-related experience, health facility-related factors, and satisfaction levels with different components of abortion services which include the availability of supply, information provision by the health worker, waiting time for service, and respect.

#### 2.5.2. Data Collection Procedures

Data collectors and supervisors were selected among individuals who are familiar with the subject of the study, and they were trained on the objectives of the study and how to conduct the interview, fill in the questionnaire, and handle questions asked by clients. Client exit interviewing was done by the data collectors. The principal investigator was also in the area till the completion of data collection for overall supervision towards ensuring data collection techniques and to check all the filled questionnaires for analysis and secure handling.

### 2.6. Data Quality Assurance

Pretest was carried out on 5% of the actual sample size (11 clients) in Adama Hospital Medical College, and the result was not included in the final report. A necessary modification was done to the data collection tool. Data collectors and data collection supervisors were provided with intensive training on the objective of the study, the contents of the questionnaires, and how to maintain the confidentiality of the study subjects. Supervision on the spot, data completeness, and clarity was checked by the principal investigators. This quality checking was done daily after data collection, and amendments were made before the next data collection measure. Data clean-up and cross-checking were done before the actual data analysis.

### 2.7. Operational Definitions

Clients' satisfaction score was categorized into two based on mean score. (1) Satisfied—those who were satisfied greater or equal to the overall sum factor mean score (≥ 78.96) of the items and. (2) Unsatisfied—those who were satisfied with less than the overall sum factor mean score (< 78.96) of the items.

### 2.8. Data Analysis and Management

The collected data were checked for completeness, and inconsistencies then coded and entered into Epi info version 7 statistical software. Then, the data was exported to Statistical Package for Social Science (SPSS) version 20 software for analysis. Twenty-six (26) variables were used to determine the overall satisfaction score. Respondents were given 5 Likert scale “Strongly Agree,” “Agree,” “Neutral,” “Disagree,” and “Strongly Disagree” response options, and responses were assigned, 5,4,3,2, and 1 points, respectively, and each respondent achieves between 26 and 130 score points.

The descriptive analysis of data was done using numerical summary measures, and the data were presented using frequency tables, figures, and graphs.

Bivariable and multivariable analysis was done to test for association between dependent and independent variables. Variables with a *P* value less than 0.25 in a bivariable binary logistic regression analysis were entered into a multivariable binary logistic regression using ENTER methods to determine factors independently associated with women's overall satisfaction.

Model fitness and multicollinearity were checked by using the Hosmer-Lemeshow goodness of fit test and the variance inflation factor (VIF). Results of logistic regression were reported as adjusted odds ratio (AOR). Those variables with a *P* value less than 0.05 with a 95% confidence interval were considered as significantly associated with the outcome variable.

### 2.9. Ethical Consideration

Ethical clearance was obtained from the ethical review committee of Adama Hospital Medical College department of public health. The official permission letter was sent to the Mojo town health office and then to the health facility in which the actual data collection was undertaken.

The purpose and importance of the study were explained, and informed consent was secured from each participant. Participants' involvement in the study was voluntary; participants who are unwilling to participate in the study and those who wish to quit their participation at any stage were informed to do so without any restriction.

## 3. Results

### 3.1. Sociodemographic and Care-Related Characteristics of the Study Participants

The data were collected from a total of two hundred fifty-five women visiting public health institutions in Mojo town for abortion services which makes the response rate 100%.

The mean age of the participants is 24.3 years (SD ± 5.2). About one hundred thirty-four (52.5%) of them were single. As to religion, 171 (67.1%) were Christian, and 206 (80.2%) were urban in residence. Concerning the educational level of participants, 73 (28.6%) were educated to college and above level, with the highest proportion of high school graduates 90 (35.5%). In terms of their occupation, 47 (18.4%) were employees, and the students constitute the highest proportion, 92 (36.1%) (Table 1).

### 3.2. Reproductive History and Health Facility Related Factor

Concerning the reproductive history of the study subjects, 248 (97.3%) were aware of abortion service availability, and only seventeen (6.7%) participants had previous abortion history. A total of 252 (98.8%) participants heard about family planning of whom 237(92.9%) had a history of contraceptive use, and natural family planning method users constitute the highest proportion 122 (47.8%). Concerning health facility-related factors, 157 (61.6%) visited the health center, 180 (70.6%) diagnosed with induced abortion, and 161 (63.1%) utilized medication for uterine evacuation (Table 2).

### 3.3. Women's Satisfaction with Abortion Service

**Figure 2:**
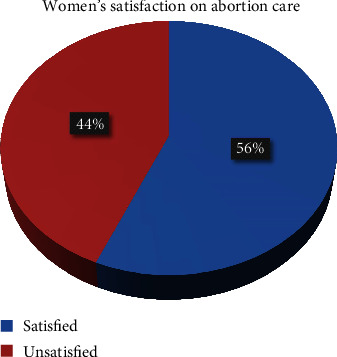
Women satisfaction with abortion service and associated factors among clients who received the service in public health facilities of Mojo town.

The study depicted that 56.5% (95% CI: 51.3, 61.7) of the clients were satisfied with the abortion care service (Figure 2). From those variables measured for the satisfaction level, greater than 70% of participants reported agreeing with greeting and accepting, respecting the client, examining with respect, and a sense of compassion from a caregiver. However, greater than 50% of participants reported disagreeing with waiting time, cleanness of rooms, lack of laboratory services, and availability of health workers (Table 3).

### 3.4. Factors Associated with Women's Satisfaction with Abortion Care

Using a *P* value less than 0.25 as criteria to select variables for binary logistic regression analysis, variables like Educational status, occupation, level of health facility, type of uterine evacuation done, and type of contraceptive used were entered into the multivariable regression model. Then, variables like age, marital status, religion, residence, type of diagnosis made, information availability on abortion services, history of previous abortion, and family planning use were dropped from the regression model. After adjusting for the variables in the model, variables statistically associated with satisfaction were educational status, occupation, type of uterine evacuation, and type of family planning method used.

Accordingly, those respondents having college and above educational level were 73% (AOR: 0.27; 95% CI: (0.14, 0.95) less likely to be satisfied when compared to those uneducated participants. However, those respondents with the occupation of the employee were 1.9 times (AOR: 1.86; 95% CI: (1.41, 2.93) more likely to be satisfied with abortion services when compared to those other occupations. Also, those respondents who utilized abortion medication for uterine evacuation were 3.9 times (AOR: 3.93; 95% CI: (1.75, 8.83) more likely to be satisfied with abortion care service when compared to those who utilized surgical methods for uterine evacuation. But respondents who are using the natural family planning method were 64% (AOR: 0.36; 95% CI: (0.08, 0.60) less likely to be satisfied with abortion care when compared to those who use other family planning methods (Table 4).

## 4. Discussion

This study has assessed the women's satisfaction and factors associated with abortion care in Mojo town public health facilities. Accordingly, 97.3% were aware of abortion service availability, and 6.7% of them had previous abortion history. Similarly, 98.8% of participants heard about family planning of whom 92.9% had a history of contraceptive use. And the study showed that 56.5% of the women were satisfied with comprehensive abortion care. From those variables measured for the satisfaction level, greater than 70% of participants reported agreeing with greeting and accepting, respecting the client, examining with respect, and a sense of compassion from a caregiver. Having college and above educational level, employee occupation, medical abortion as a type of uterine evacuation, and natural method of family planning users are factors identified as having association with comprehensive abortion care.

This finding is lower compared to a study conducted in the Jimma zone which showed 76.3% [21] and a study conducted in the Gurage zone which showed 83.5% [16]. But this finding is higher than the study conducted in Tigray (40.6%) and Northwest Ethiopia (25%), where the clients were satisfied with the service [6, 23]. The possible reason for these differences in satisfaction with the service may be due to differences in the study setting and study period. Moreover, disparities in the attitude and skill of healthcare workers, lack of coordination, and negligence of healthcare providers are among the possible factors for this discrepancy.

We found that educational level showed significant associations with women's satisfaction with abortion care services. The odds of satisfying abortion services among those having college and above educational level were lower when compared to those not having an education. The result of this study is similar to the study done in Tigray health facilities and New York [13, 24]. This could be as people learn more; they also expect more from the service provider to be satisfied and communication gap between health care workers and clients, which means the less educated client may not ask their interest for better service. And educated women have sufficient information as the result of their residency (educated women are more likely to live in urban areas), have better access to different health facilities, and have more media exposure which may increase their expectations. The occupation of women is also another significant factor for this study. Employed women were more likely to be satisfied than those in other occupations. This finding is in line with the study conducted in Jimma town and Indonesian women [21, 25]. The possible reason may be that employed women have a higher income, can buy post-abortion therapy drugs in the pharmacy, and have better communication with the health care providers which may help them to understand the scarcity of resources occurring in the health facility to deliver the procedure accordingly.

According to the current study, clients who utilized medication abortion are more likely to be satisfied than those who utilized surgical procedures for uterine evacuation. This result is different from the findings conducted in Addis Ababa [22]. The reason may be the painful and irritating characteristics of the surgical procedure and since the medication is used for induced abortion; they may come to the facilities with very low expectations anticipating that they may not be well treated by the providers due to interference with their pregnancies [26].

Respondents who are using natural family planning methods were less likely to be satisfied with abortion services when compared to those who used other types of family planning methods. This finding is consistent with the study conducted in Tigray and Jimma [27, 28]. The possible justification for this difference may be a lack of information on abortion services and unfamiliarity with the hospital environment.

### 4.1. Limitations of the Study

Causal inferences may not be established due to the study design used (cross-sectional) and the data collected at specified time points. The study was conducted in a single town with fewer health facilities and a sample size, which may have affected the generalizability of the results. Another limitation of the current study was the lack of using mixed study designs.

## 5. Conclusion

This study showed as more than half of the women were satisfied with the abortion service they had. Participants mentioned certain factors for their dissatisfaction as waiting time, cleanness of rooms, lack of laboratory service, and the availability of service providers. The predictors of women's satisfaction with abortions service identified in this study were the occupation of an employee, type of uterine evacuation with a medication abortion, and natural family planning methods.

### 5.1. Recommendations

Based on the findings of the current study, the authors forwarded the following recommendations.

We recommend that the responsible authorities—regional, zonal, and Woreda health office; health care providers; NGOs working on this area; policymaker; and interested body—has to discuss this issue to enhance the client satisfaction gap and to develop a system to control client satisfaction and control factors that affect client satisfaction. The health care providers should provide services that meet the client's needs, such as having a choice of services, receiving reliable and complete information, receiving technically competent treatment, having good interactions with providers, having a quality of care, and a constellation of relevant services. They should work on the improvement of waiting time of the client, cleanness of rooms, and timely availability at the workplace.

Furthermore, any interested body can investigate a qualitative study to prove and support or disprove the findings.

## Figures and Tables

**Table 1:** Sociodemographic characteristics of participants receiving abortion care in public health facilities of Mojo town.

Variables	Frequency	Percent (%)
Age (in years)		
15-19	59	23.4
20-24	85	33.3
25-34	100	39.2
≥35	11	4.3
Marital status		
Single	134	52.5
Married	108	42.4
Other^∗^	13	5.1
Religion		
Christian	211	82.7
Muslim	44	17.3
Residence		
Urban	206	80.8
Rural	49	19.2
Educational level		
No education	32	12.6
Primary education	60	23.5
Secondary education	90	35.3
College and above	73	28.6
Occupation		
Employee	47	18.4
Housewife	30	11.8
Merchant	59	23.1
Student	92	36.1
Other^∗∗^	27	10.6

Other^∗^ = divorced, widowed; other^∗∗^ = daily laborer, servant, and no job.

**Table 2:** Distribution of participants receiving abortion care by their previous reproductive health experience and health facility-related factors in Mojo town.

Variables	Frequency	Percent
Information about the availability of abortion service		
No	7	28.4
Yes	248	71.6
History of previous abortion		
No	238	68.5
Yes	17	31.5
Heard about family planning		
No	3	5.2
Yes	252	94.8
Ever used the family planning method		
No	18	45.5
Yes	237	54.5
Type of family planning method used		
Natural	122	47.8
Pills	74	29.0
Injectable	49	19.2
Other^+^	10	3.9
Type of health facility		
Health center	157	61.6
Hospital	98	38.4
Type of diagnosis		
Induced abortion	180	70.6
Postabortion care	75	29.4
Type of uterine evacuation		
Medical	161	63.1
Surgical	94	36.9

Other^+^ = condom, implants, IUCD.

**Table 3:** Women satisfaction-related characteristics of participants in public health facilities of Mojo town.

Variables	Level of satisfaction		
	Agree (%)	Neutral (%)	Disagree (%)
Healthcare providers greet and accept clients well	194 (76.1)	18 (7.0)	43 (16.9)
Health care provider respected the client	187 (73.3)	22 (8.6)	46 (18.0)
The provider gives service with a sense of compassion	182 (71.4)	24 (9.4)	49 (19.2)
Provider facilitates mutual understanding for the client	165 (64.7)	43 (16.9)	47 (18.4)
Provider listens to client ideas and creates trust in service	166 (65.1)	36 (14.1)	53 (20.8)
The provider is cooperative in service provision	158(62.0)	48 (18.8)	49 (19.2)
Provider involve in the discussion made for service	146 (57.2)	45 (17.6)	64 (25.1)
The provider gives adequate information on the service provided	159 (62.4)	38 (14.9)	58 (22.7)
The provider gives service without discrimination	157 (61.6)	66 (25.9)	32 (12.5)
Confidentiality is secured on service provided	142 (55.7)	77 (30.2)	36 (14.1)
Privacy is secured on service provided	154 (60.4)	43 (16.9)	58 (22.7)
Medical equipment used for service is modern	159 (62.4)	63 (24.7)	33 (12.9)
The provider had enough technical skills for the service	142 (55.7)	82 (32.2)	31 (12.2)
The provider examines the client with respect	183 (71.8)	18 (7.0)	54 (21.2)
The provider explained the procedure done for the client	156 (61.2)	14 (5.5)	85 (33.3)
The provider gives the necessary help to control the pain	128 (50.2)	98 (38.4)	29 (11.4)
The provider gives the necessary advice to the client	163 (63.9)	34 (13.3)	58 (22.7)
There is a comfortable waiting room	106 (41.7)	60 (23.5)	89 (34.9)
The service delivery area is clean	94 (36.9)	26 (14.1)	135 (52.9)
The service delivery area is ventilated	188 (46.3)	37 (14.5)	100 (39.2)
The service delivery area is suitable for the service	102 (40.0)	47 (18.4)	106 (41.6)
Waiting time for the service is acceptable	85 (33.3)	30 (11.8)	140 (54.9)
The working hour of the health facility is suitable for the service	135 (52.9)	26 (10.2)	94 (36.9)
Clients get laboratory service easily	94 (36.9)	33 (12.9)	128 (50.2)
Clients get drugs easily	118 (46.3)	41 (16.7)	96 (37.6)
Service providers are available at the workplace	98 (38.4)	25 (9.8)	132 (51.8)

**Table 4:** Result of bivariable and multivariable analysis on the association between the study variables among respondents in Mojo town.

Variable	Satisfaction with abortion service		AOR, 95% CI
	Satisfied (%)	Unsatisfied (%)	
Age category			
15-19	35 (13.7)	24 (9.4)	0.10 (0.01, 1.41)
20-24	53 (20.8)	32 (12.5)	0.17 (0.02, 2.10)
25-34	51 (20.0)	49 (19.2)	0.18 (0.02, 1.68)
> =35	5 (2.0)	6 (2.4)	1.00
Marital status			
Single	77 (30.2)	57 (22.3)	2.74 (0.31, 2.42)
Married	60 (23.5)	48 (18.8)	3.46 (0.46, 2.62)
Other	7 (2.8)	6 (2.4)	1.00
Religion			
Christian	120 (47.1)	91 (35.7)	1.71 (0.75, 3.90)
Muslim	24 (9.4)	20 (7.8)	1.00
Residence			
Urban	114 (44.7)	92 (30.1)	0.93 (0.39, 2.21)
Rural	30 (11.8)	19 (7.4)	1.00
Educational level			
No formal education	23 (9.0)	9 (3.5)	1.00
Primary	46 (18.0)	14 (5.5)	1.51 (0.51, 4.52)
Secondary	58 (22.8)	32 (12.5)	0.82 (0.26, 2.61)
College and above	17 (6.7)	56 (22.0)	0.27 (0.14, 0.95)^∗^
Occupation			
Employee	40 (15.7)	7 (2.7)	1.86 (1.41, 2.93)^∗^
Housewife	21 (8.2)	9 (3.5)	1.79 (0.48, 6.71)
Merchant	38 (14.9)	21 (8.2)	1.30 (0.39, 4.305)
Student	59 (23.2)	33 (13.0)	0.60 (0.17, 2.14)
Other	19 (7.5)	8 (3.1)	1.00
Level of health facility			
Health center	101 (39.6)	56 (21.9)	1.26 (0.34, 1.183)
Hospital	43 (16.9)	50 (19.6)	1.00
Type of uterine evacuation			
Medical abortion	100 (39.2)	61 (23.9)	3.93 (1.75, 8.83)^∗^
Surgical	44 (17.3)	50 (19.6)	1.00
Type of family planning method used			
Natural	45 (17.6)	77 (30.2)	0.36 (0.08, 0.60)^∗^
Pills	54 (21.2)	20 (7.8)	1.44 (0.40, 5.24)
Injection	40 (15.7)	9 (3.5)	0.70 (0.19, 2.56)
Other	5 (2.0)	5 (2.0)	1.00

^∗^ = *P* value < 0.05.

## Data Availability

All data used in the current study are cited well in the document.

## Abbreviations

AOR: Adjusted odds ratio
CAC: Comprehensive abortion care
CDC: Center for disease control
CI: Confidence interval
COR: Crude odd ratio
D&C: Dilatation and curettage
EDHS: Ethiopian demographic health survey
FMOH: Federal Minister of Health
IPAS: International pregnancy advisory service
IPPF: International Planned Parenthood Federation
LNMP: Last Normal menstrual period
MVA: Manual vacuum aspiration
OR: Odds ratio
PAC: Post-abortion care
SAC: Safe abortion care
SPSS: Statistical package for social science
USAID: United States agency for international development
VIF: Variance inflation factor
WHO: World Health Organization.
